# Macrophage-derived small extracellular vesicles promote biomimetic mineralized collagen-mediated endogenous bone regeneration

**DOI:** 10.1038/s41368-020-00100-6

**Published:** 2020-11-30

**Authors:** Anqi Liu, Shanshan Jin, Cuicui Fu, Shengji Cui, Ting Zhang, Lisha Zhu, Yu Wang, Steve G. F. Shen, Nan Jiang, Yan Liu

**Affiliations:** 1 Department of Oral and Maxillofacial Surgery, Ninth People’s Hospital, Shanghai Jiao Tong University School of Medicine, Shanghai Key Laboratory of Stomatology, Shanghai, China; 2 Laboratory of Biomimetic Nanomaterials, Department of Orthodontics, Peking University School and Hospital of Stomatology, National Engineering Laboratory for Digital and Material Technology of Stomatology, Beijing Key Laboratory of Digital Stomatology, Beijing, China; 3 Department of Oral Biochemistry, Academic Centre for Dentistry Amsterdam (ACTA), University of Amsterdam (UvA) and Vrije Universiteit Amsterdam (VU), Gustav Mahlerlaan 3004, Amsterdam, The Netherlands; 4 Shanghai University of Medicine and Health Sciences, Shanghai, China; 5 Central Laboratory, Peking University School and Hospital of Stomatology, National Engineering Laboratory for Digital and Material Technology of Stomatology, Beijing Key Laboratory of Digital Stomatology, Beijing, China

**Keywords:** Biomedical engineering, Mesenchymal stem cells

## Abstract

Macrophages play an important role in material-related immune responses and bone formation, but the functionality of macrophage-derived extracellular vesicles (EVs) in material-mediated bone regeneration is still unclear. Here, we evaluated intracellular communication through small extracellular vesicles (sEVs) and its effects on endogenous bone regeneration mediated by biomimetic intrafibrillarly mineralized collagen (IMC). After implantation in the bone defect area, IMC generated more neobone and recruited more mesenchymal stem cells (MSCs) than did extrafibrillarly mineralized collagen (EMC). More CD63^+^CD90^+^ and CD63^+^CD163^+^ cells were detected in the defect area in the IMC group than in the EMC group. To determine the functional roles of sEVs, extracellular vesicles from macrophages cultured on different mineralized collagen were isolated, and they showed no morphological differences. However, macrophage-derived sEVs in the IMC group showed an enhanced Young’s modulus and exerted beneficial effects on the osteogenic differentiation of bone marrow MSCs by increasing the expression of the osteoblastic differentiation markers BMP2, BGLAP, COL1, and OSX and calcium nodule formation. Mechanistically, sEVs from IMC-treated macrophages facilitated MSC osteogenesis through the BMP2/Smad5 pathway, and blocking sEV secretion with GW4869 significantly impaired MSC proliferative, immunomodulative and osteogenic potential. Taken together, these findings show that macrophage-derived sEVs may serve as an emerging functional tool in biomaterial-mediated endogenous bone regeneration.

## Introduction

Immune homeostasis is essential for successful bone regeneration driven by biomaterial scaffolds.^1^ Host-biomaterial reactions were previously associated with rejection; to date, innate immune effector cells, most notably macrophages, have been identified as important mediators and instructors during scaffold remodeling and tissue regeneration.^2–4^ Immunoengineering via the development of ‘immune-interactive’ smart biomaterials has emerged as an effective strategy to improve bone regenerative outcomes. This strategy utilizes biomaterial physicochemical modifications, including surface topography and chemistry, inorganic components and biomimetic bone architecture, to offer an appropriate microenvironment for immune responses, host cell recruitment and differentiation.^5–8^

Macrophages are remarkably plastic and can tactically shift to a pro-inflammatory M1 phenotype, or alternatively, an anti-inflammatory M2 phenotype by adapting to the local microenvironment.^9^ The balance of the two macrophage phenotypes plays an important role in pathogen phagocytosis, apoptotic cell clearance, and tissue remodeling.^10^ Monocyte/macrophage depletion impairs osteoblastic differentiation and bone regeneration.^5,11^ In contrast, enhanced monocyte/macrophage recruitment induced by an agonist of sphingosine-1-phosphate type 1 receptor facilitates bone repair.^12^ Other immune-modulatory approaches to enhance bone regeneration include a switch to M2 macrophage polarization and sequential M1-to-M2 macrophage phenotype transition.^13^

Bone formation and remodeling are closely related to the interactions between polarized macrophages and mesenchymal stem cells (MSCs), which can occur via cell–cell contact and/or via cytokine production.^14^ Extracellular vesicles (EVs) are particles that are naturally released from cells and play an important role in cellular communication.^15^ Small EVs (sEVs) are a subtype of EVs with diameters less than 200 nm, including exosomes and macrovesicles.^16^ Macrophage-derived sEVs have been reported to communicate with neighboring cells by modulating cytokine and miRNA levels to relieve inflammatory responses.^17^ In our previous study, we successfully fabricated hierarchical intrafibrillarly mineralized collagen (IMC) by mimicking the surface chemistry and hierarchical topography of natural bone and proved that IMC possesses the capacity to recruit host MSCs and promote endogenous bone regeneration via immunomodulation of M2 macrophage polarization through interleukin-4.^5^ However, whether the paracrine mechanism mediated by EVs also plays an important role in IMC-mediated endogenous bone regeneration remains unclear. Therefore, we investigated the regulatory role of macrophage-derived sEVs in IMC-mediated bone regeneration. The results presented herein may provide new insights into the mechanisms by which the local microenvironment affects material-mediated bone regeneration.

## Results

### Promoting endogenous bone regeneration and MSC recruitment with biomimetic mineralized collagen

**Fig. 1:**
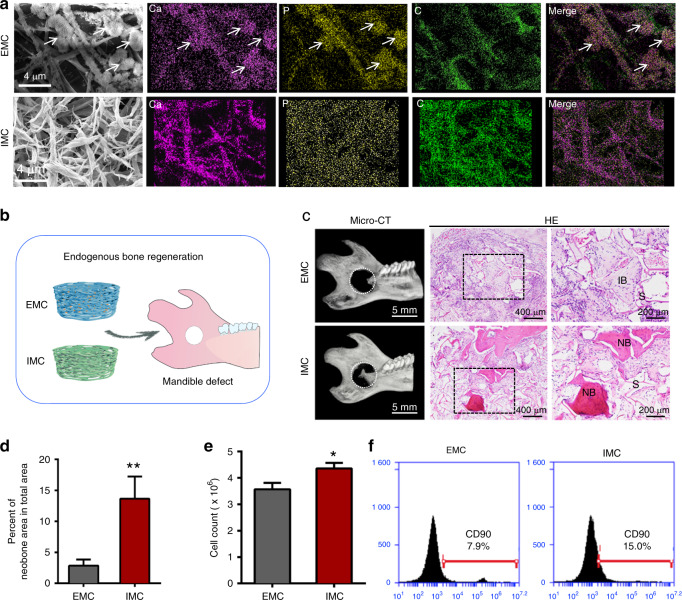
Promoting endogenous bone regeneration and MSC recruitment with IMC in vivo. **a** SEM and EDS mapping of IMC and EMC. Arrows: apatite clusters. **b** Schematic diagram of a mandible defect and scaffold implantation. **c** Representative micro-CT and H&E staining images of the engineered bone with EMC and IMC at 2 weeks post implantation. NB new bone, IB immature bone, S scaffold. **d** Semiquantification of the neobone area. **e** Flow cytometry analysis of cell counts with EMC and IMC at 1 week post implantation. **f** Flow cytometry analysis of CD90^+^ cell numbers in the EMC and IMC groups

Biomimetic mineralized collagen was fabricated according to our previously published procedures,^18,19^ and its typical microstructure and element content were observed by scanning electron microscopy (SEM) and energy dispersive X-ray spectroscopy (EDS) (Fig. 1a). The IMC exhibited a pronounced fibrous texture, while flower-like apatite clusters (1.65 μm ± 0.30 μm) were randomly deposited around EMC fibers. The presence of intrafibrillar apatite (Ca/P = 1.53) in IMC was confirmed using EDS mapping, which showed a similar distribution of Ca (indicating apatite) and C (indicating collagen) and a homogeneous distribution of Ca inside collagen fibers. By contrast, the distribution of Ca and C was opposite in EMC, with an area of elevated Ca concentration corresponding to an area of reduced C concentration. To examine the bone regeneration potential of IMC and EMC, scaffolds without loaded exogenous cells or cytokines were transplanted into critical-sized rat mandible defects (Fig. 1b). After 2 weeks of transplantation, more neobone was formed with osteocytes embedded in the IMC group than in the EMC group (Fig. 1c, d). Similar to our previous studies,^5,18,19^ no neobone formation was detected in the defect area without any implants (data not shown). Flow cytometry analysis of neotissue showed that more cells were recruited to the defect area by IMC, especially CD90 (a surface marker of MSCs)-positive cells (Fig. 1e, f). CD90^+^ cells accounted for 15% of the cells in the IMC group and only 7.9% of the cells in the EMC group. Consistent with our previous findings, these data indicate that IMC could promote endogenous bone regeneration and MSC recruitment.^5,18^

### Increasing extracellular vesicles and M2-polarized macrophages with IMC during endogenous bone regeneration

**Fig. 2:**
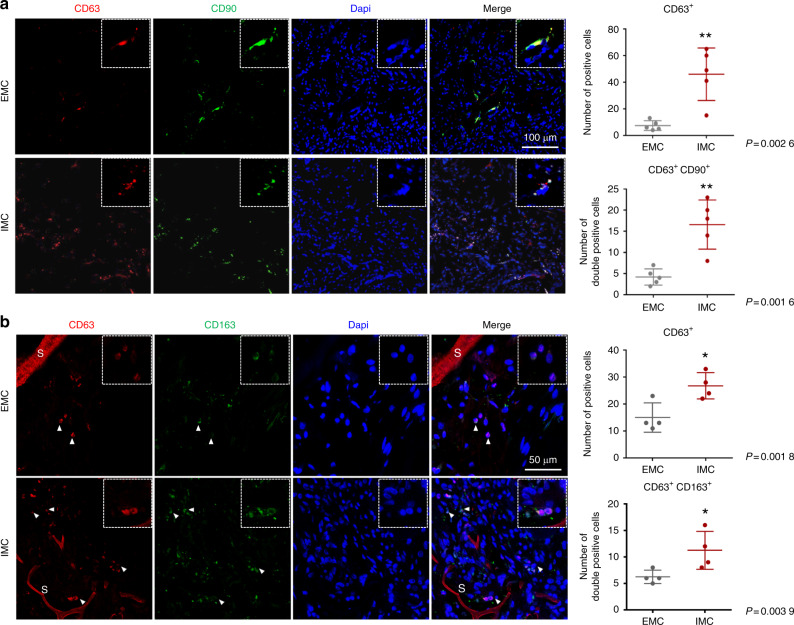
Representative immunofluorescence staining of CD63, CD90, and CD163 in defect areas. **a** Immunofluorescence staining of CD63 and CD90 and semiquantification of positively stained cells. **b** Immunofluorescence staining of CD63 and CD163 and semiquantification of positively stained cells. *P < 0.05 vs EMC, **P < 0.01 vs EMC

Previously, we successfully demonstrated that biomimetic IMC promoted MSC osteogenic differentiation by regulating M2 macrophage polarization during bone regeneration.^5^ However, whether the paracrine mechanism mediated by EVs participates in IMC-mediated endogenous bone regeneration remains unclear. Immune cells and MSCs can transfer information by secreting EVs during tissue healing and regeneration.^20^ Here, we found through immunofluorescence staining that CD63, a classic surface marker of EVs, was highly expressed in the neotissue generated by IMC (Fig. 2a). Interestingly, CD63 and CD90 were found to be highly coexpressed in the IMC group, which had four times more CD63^+^CD90^+^ cells than the EMC group. Similar to our previous results, IMC also promoted M2 macrophage polarization, with more CD163^+^ cells than observed in the EMC group (Fig. 2b). CD63 and CD163 were found to be highly coexpressed surrounding the IMC scaffold, with two times more CD63^+^CD163^+^ cells than observed in the EMC group. Overall, IMC could promote the secretion of EVs, which might participate in IMC-mediated endogenous bone regeneration.

### Identification and characterization of extracellular vesicles derived from macrophages seeded on different types of mineralized collagen in vitro

**Fig. 3:**
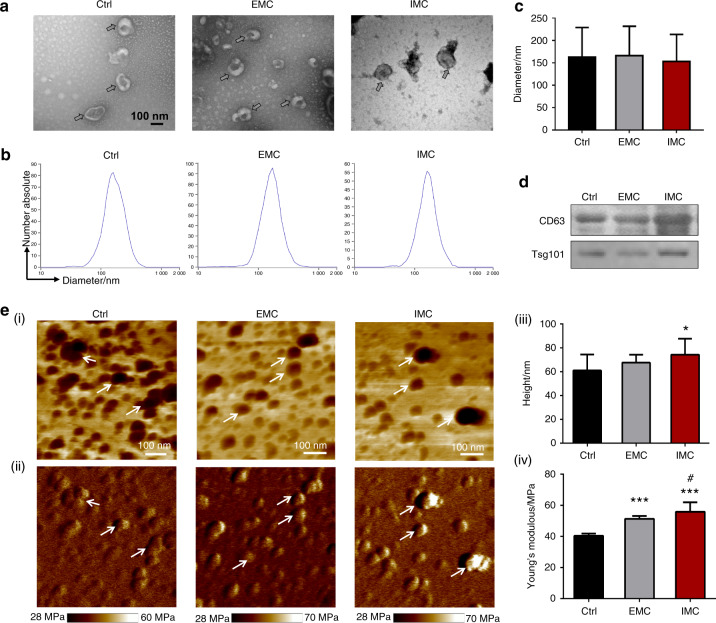
Characterization of extracellular vesicles (EVs) derived from macrophages seeded on mineralized collagen in vitro. **a** TEM morphology of extracellular vesicles. **b** Corresponding particle size and distribution analysis of the samples in **a**. **c** Semiquantification of extracellular vesicle diameter. **d** Western blotting of EV surface markers. **e** (i) AFM phase images of EVs; (ii) Corresponding modulus mapping images of EVs; (iii) Semiquantification of EV height; (iv) Semiquantification of EV modulus. Arrows: EVs; Ctrl: normal macrophage-derived sEVs. *P < 0.05 vs Ctrl, ***P < 0.001 vs Ctrl, ^#^*P* < 0.05 vs EMC

To determine whether mineralized collagen affects extracellular vesicle functions and participates in bone regeneration, we isolated and characterized EVs from THP-1-derived macrophages cultured on IMC (IMC-sEVs) and EMC (EMC-sEVs) scaffolds. EVs secreted from macrophages on regular culture plates were used as a control (Ctrl-sEVs). In brief, EVs were harvested from culture supernatants by ultracentrifugation and identified by transmission electron microscopy (TEM), atomic force microscopy (AFM), and Western blotting. Representative TEM images demonstrated that the collected EVs bore spherical and membrane-encapsulated structures, which are typical morphologies (Fig. 3a). Nanoparticle tracking analysis indicated the presence of ~150 nm cellular particles, and no statistically significant difference was found among groups (Fig. 3b, c). Western blot images showed the expression of the EV-specific markers CD63 and Tsg101 (Fig. 3d). To further detect the architecture of EVs under native conditions, freshly isolated EVs without staining were further observed by AFM (Fig. 3e).^21^ AFM phase images revealed a round morphology of 40–100 nm EVs with a prominent phase contrast. The EV height observed by AFM was smaller than the diameter observed by TEM, which might be because the fresh soft nanoparticles collapsed on the hard mica substrate. However, the EVs were stable without lysis and showed some aggregation without intervesicular fusion. Some EVs displayed a characteristic ring-like trilobed structure, which might be attributed to proteins and/or mRNA enclosed inside the lipid membrane.^21^ Furthermore, the Young’s modulus of IMC-EVs (~56 MPa) and EMC-EVs (~51 MPa) was much higher than that of Ctrl-EVs (~40 MPa). These results indicated that EVs isolated from macrophages coated on different mineralized collagen scaffolds exhibited the physical characteristics of sEVs and were not significantly different in appearance and diameter.^22^ Force measurements of macrophage-derived sEVs revealed a high Young’s modulus, which is consistent with a previous study.^23^ These results unveil an important role of natural membrane vesicles as robust nanocontainers for applications.

### Biological functions of MSCs stimulated with IMC-sEVs

**Fig. 4:**
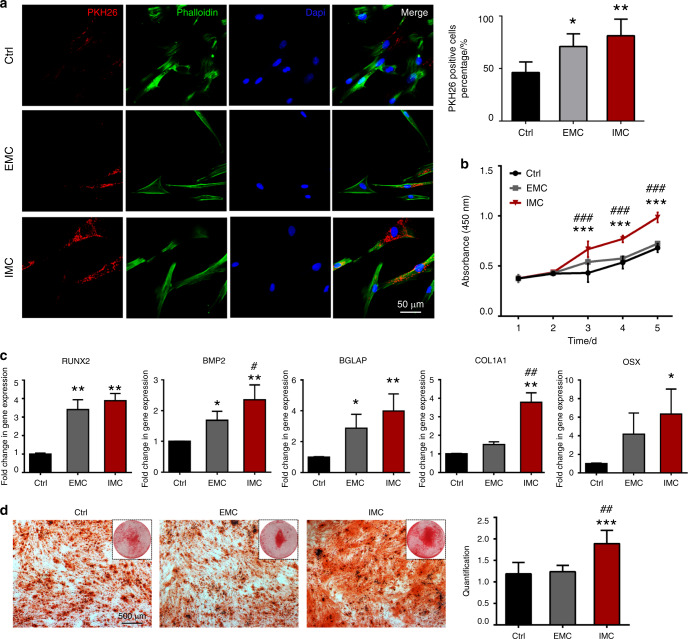
Biological functions of MSCs stimulated with IMC-sEVs. **a** Representative immunofluorescence staining of the EV-labeling marker PKH26. **b** Semiquantification of positively stained cells. **c** MSC proliferation ability after stimulation with macrophage-derived sEVs. **d** Relative mRNA expression levels of Runx2, BMP2, BGLAP, COLA1, and OSX in MSCs stimulated with macrophage-derived sEVs for 14 days. **e** Alizarin Red S staining of MSCs stimulated by macrophage-derived sEVs for 14 days. *P < 0.05 vs Ctrl; **P < 0.01 vs Ctrl; ***P < 0.01 vs Ctrl; ^#^*P* < 0.05 vs EMC; ^##^*P* < 0.05 vs EMC; ^###^*P* < 0.05 vs EMC

Fundamental cellular processes, including proliferation and differentiation, can be influenced by EVs that carry informative cargo.^24^ To determine whether sEVs functionally affected MSC biological functions, human bone marrow MSCs were cultured with sEVs (1 μg·mL^−1^) from THP-1 cells cultured on different mineralized collagen scaffolds. The uptake of sEVs by MSCs was first investigated through immunofluorescence staining (Fig. 4a, b). The sEVs were labeled with PKH26 and incubated with MSCs for 4 h. As shown in Fig. 4a, sEVs from all the THP-1 cells were incorporated into MSCs. Interestingly, significantly higher red fluorescence intensity and ~81.2% PKH26^+^ cells were observed in the IMC group, suggesting that MSCs preferentially endocytosed sEVs from THP-1 cells cultured on IMC. Next, the proliferation ability of MSCs was examined after stimulation with macrophage-derived sEVs. MSCs in all the groups grew slowly at the beginning of the experiment. From day 3, cells treated with IMC-sEVs started to grow more robustly than those treated with regular macrophage-derived sEVs and even more robustly than those treated with EMC-sEVs (*P* < 0.001, Fig. 4c). To further verify the osteogenic function of sEVs, human bone marrow MSCs were cultured in osteogenic medium supplemented with sEVs (1 μg·mL^−1^). After 14 days, the mRNA expression levels of osteogenic differentiation markers runt-related transcription factor 2 (RUNX2), bone morphogenetic protein 2 (BMP2), and bone gamma-carboxyglutamate protein (BGLAP) were highly upregulated with both EMC-sEV and IMC-sEV stimulation (Fig. 4d). In particular, IMC-sEVs showed a better osteogenic induction effect than EMC-sEVs. The mRNA expression levels of the osteogenic differentiation markers alpha-1 type I collagen (COL1A1) and Osterix (OSX) were only increased when applying IMC-sEVs. In addition, following 14 days of osteogenic induction, MSCs differentiated into osteoblasts that formed more mineral nodules with IMC-sEV stimulation (Fig. 4e). Collectively, IMC-sEVs promoted the endocytosis, proliferation, and osteogenic differentiation of MSCs.

### MSC osteogenesis stimulated with IMC-sEVs through the BMP2/Smad5 pathway

**Fig. 5:**
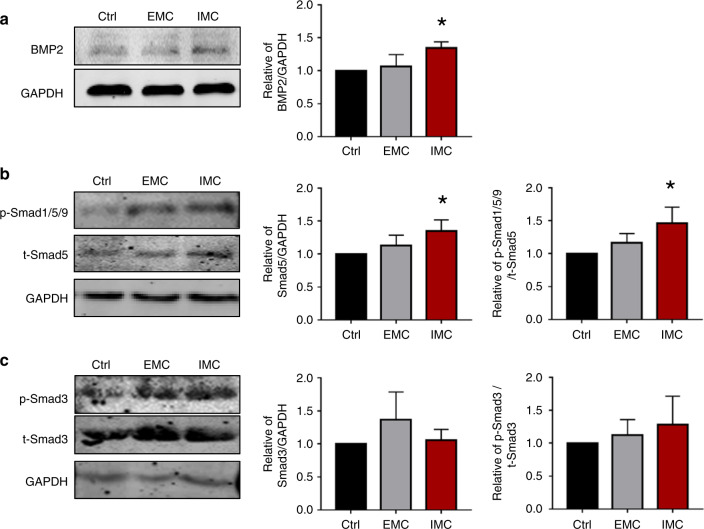
Western blot analysis of BMP2 (**a**), Smad1/5/9 (**b**), and Smad3 (**c**) in MSCs stimulated with macrophage-derived sEVs for 14 days. *P < 0.05 vs Ctrl

To reveal the underlying mechanisms of MSC osteogenesis, the protein levels of MSCs among groups were further examined by Western blotting. The BMP signaling pathway regulates bone formation, and canonical BMP signaling through Smad1/5 is required for endochondral bone formation.^25^ Here, BMP2 expression was significantly increased with IMC-sEV stimulation (Fig. 5a). Since sEVs incorporate proteins, DNAs, RNAs and lipids, we probed the BMP2 protein in the macrophage-derived sEVs to determine whether the BMP2 change in BMSCs was caused directly by sEV proteins. As shown in Fig. S1, no BMP2 was detected in sEVs derived from macrophages cultured on either plates (Ctrl) or EMC/IMC scaffolds. The Smad1/5/9 signaling pathway could be activated by BMP2 and induced downstream Runx2 expression. We examined Smad5 expression and found obvious upregulation in the IMC group (Fig. 5b). By contrast, the Smad3 protein level showed no significant change among the different groups (Fig. 5c). As a result, the promotion of MSC osteogenesis is mainly mediated by activation of the BMP2/Smad5 pathway but not inhibition of Smad3 signaling.

**Fig. 6:**
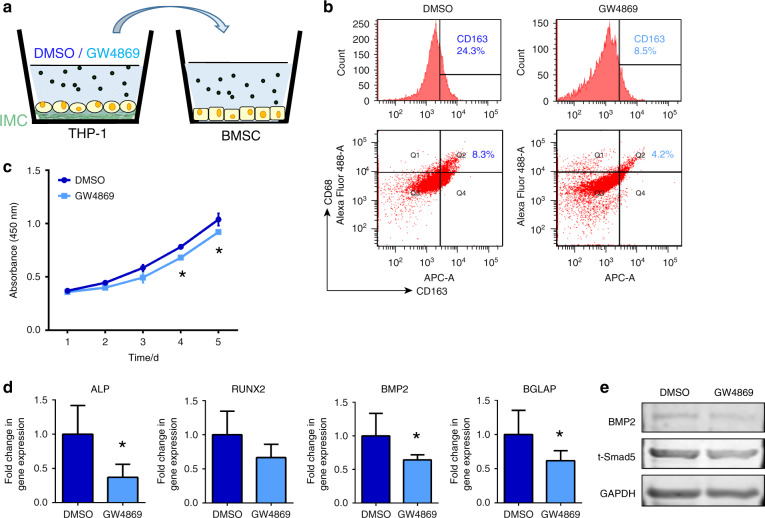
Suppression of MSCs proliferative, immunomodulative and osteogenic potential after blocking EV secretion. **a** Schematic diagram. **b** Flow cytometry analysis of CD163^+^ and CD68^+^ cell numbers after blocking EV secretion. **c** Impaired MSC proliferation rate after blocking EV secretion. **d** Decreased mRNA levels of ALP, RUNX2, BMP2, and BGLAP in MSCs when treating cells with GW4869 conditioned medium. **e** Inhibition of BMP2 and Smad5 protein levels in MSCs when treating cells with GW4869 conditioned medium. *P < 0.05 vs DMSO

To further clarify the contribution of sEVs, we applied 10 μmol·L^−1^ GW4869 to block the secretion of sEVs from macrophages cultured on IMCs. Equal amounts of DMSO were added to macrophage culture medium as a control (Fig. 6a). After blocking secretion, CD163-positive cells decreased from 24.3% to 8.5%, and the CD163^+^CD68^+^ cell percentage dropped from 8.3% to 4.2%, indicating that the polarization of M2 macrophages was influenced by sEVs secretion (Fig. 6b). Next, we treated MSCs with DMSO or GW4869 conditioned medium and found that the proliferation rate was significantly reduced when sEVs were absent (Fig. 6c). The osteogenesis ability of MSCs was diminished as well, as revealed by suppression of bone-related genes, such as alkaline phosphatase (ALP), RUNX2, BMP2, and BGLAP, when treating cells with GW4869 conditioned medium (Fig. 6d). Furthermore, the protein levels of BMP2 and Smad5 were inhibited (Fig. 6e). Collectively, these results indicated that macrophage-derived sEVs in the IMC group are capable of promoting MSC osteogenic differentiation via BMP2/Smad5 signaling.

## Discussion

EVs are lipid bilayer nanovesicles that are secreted by most cell types and control intercellular communication. Increasing data suggest that macrophages could build an optimal microenvironment to reduce inflammation and promote osteogenesis through a paracrine mechanism.^26^ We have previously proven that IMC, as a biomimetic bone scaffold, possesses the capacity to recruit host MSCs and promote endogenous bone regeneration via immunomodulation of M2 macrophage polarization through cytokine production.^5^ However, whether the paracrine mechanism also plays an important role in IMC-mediated endogenous bone regeneration remains unclear. In this study, we found that IMC-sEVs were involved in IMC-facilitated endogenous bone regeneration in vivo and demonstrated that IMC-modified macrophages could secrete functional sEVs promoting MSC osteogenesis through the BMP2/Smad5 pathway. Therefore, macrophage-derived sEVs may serve as an emerging functional tool for biomaterial-mediated endogenous bone regeneration.

MSC-derived sEVs have been well studied and identified as a novel therapeutic strategy in tissue engineering and regenerative medicine.^27^ Effective bone regeneration outcomes have been achieved via the combined use of MSC-derived sEVs and biomaterials.^28^ Although macrophages play an important role in biomaterial-related immune reactions in tissue engineering, very little research has been conducted on the role of macrophage-derived sEVs in bone regeneration. Li et al.^29^ demonstrated that sEVs, such as exosomes, secreted by macrophages could inhibit inflammation and accelerate diabetic wound healing. Wei et al.^30^ investigated the possibility of integrating exosomes derived from BMP2-activated macrophages into titanium nanotubes as important regulatory molecules to enhance osteogenesis. Here, we found that IMC-sEVs facilitated MSC osteogenesis and might play an important role in biomaterial-mediated endogenous bone regeneration. Moreover, we also detected the underlying mechanisms of IMC-sEVs in the osteogenesis of MSCs. The protein expression levels of BMP2 and Smad1/5/9 were significantly increased in MSCs stimulated with IMC-sEVs, while no statistically significant difference in Smad3 was detected among the groups. Functionally, blockade of EV secretion by GW4869 significantly impaired MSC osteogenesis. Smad5 is one of the classical proteins of the osteogenic pathway. Xu et al.^31^ confirmed that Smad5 is a direct downstream target of miR-128-3p, thus suggesting that the protective function of MSC-derived exosomes on osteogenic differentiation and fracture healing may vary according to differential expression of miRNAs.

The biofunctions of EVs rely on their capacity to communicate with recipient cells and to deliver components (i.e., lipids, proteins, and nucleic acids) to these cells.^32,33^ Zhou et al.^34^ have shown that exosomes from tumor-associated macrophages regulate the immune microenvironment by transferring miRNAs. Similarly, hypothalamic stem cells control ageing speed partially through the release of exosomal miRNAs.^35^ Here, more CD63^+^CD90^+^ and CD63^+^CD163^+^ cells existed in the neotissue generated by IMC, suggesting that IMC promoted macrophage-derived extracellular vesicle secretion and interactions between CD63^+^ EVs and CD90^+^ MSCs or CD163^+^ M2-polarized macrophages. The in vitro experiments showed that IMC-sEVs promoted MSC endocytosis, proliferation, and osteogenic differentiation, while the morphology and diameter of IMC-sEVs and EMC-sEVs were similar. Thus, the essential contents packaged in IMC-sEVs and their role in MSC functions need further investigation.

The biomaterial-mediated bone regeneration process involves complex interactions between immune cells and MSCs and MSCs themselves. Paracrine mechanisms mediated by secreted factors contribute to the bone regenerative function of MSCs.^36^ MSC-derived sEVs have been proven to be key contributors to MSC paracrine effects, including immunomodulation, migration, differentiation, and angiogenesis.^37^ Furthermore, sEVs from differentiating MSCs have osteoinductive capacities and could induce osteogenic differentiation of naïve MSCs.^38,39^ In the present study, we mainly focused on the functional role of macrophage-derived sEVs in MSC osteogenesis and IMC-mediated endogenous bone regeneration. Our future work needs to investigate the potential role of MSC-derived sEVs in the immunomodulation, migration, and osteogenic differentiation of MSCs in biomaterial-mediated bone regeneration.

Overall, our study evaluated the potential role of macrophage-derived sEVs in biomimetic mineralized collagen-mediated endogenous bone regeneration in vivo and in vitro. Macrophage-derived sEVs were involved in IMC-mediated endogenous bone regeneration in vivo, and IMC-sEVs facilitated MSC osteogenesis through the Smad/1/5/9 pathway. Within the limitations of this study, we conclude that macrophage-derived sEVs may serve as an emerging functional tool in biomaterial-mediated endogenous bone regeneration.

## Materials and methods

### Scaffold preparation and characterization

Biomimetic IMC was fabricated according to our previously published procedures.^18^ Briefly, tropocollagen solution (Corning) was continually dropped into a dialysis flask (3500 Da), which was soaked in a mineralization solution containing 136.9 mM NaCl, 2.7 mM KCl, 8.3 mM Na_2_HPO_4_, 1.25 mM K_2_HPO_4_·3H_2_O, 0.25 mM poly(acrylic acid), 3.08 mM Na_3_N, and 0.2 g/mL white Portland cement (Lehigh Cement Co.) for 7 days. Without poly(acrylic acid) in the solution, EMC formed. To create three-dimensional sponge-like scaffolds, fibrillized collagen was poured into 48-well polystyrene culture plates, frozen for 24 h at −30 °C, and lyophilized for use. The microstructure and element content of scaffolds were observed by SEM and EDS coupled to SEM (Hitachi S-4800).

### Animal surgery and tissue preparation

Critical defects with a diameter of 5 mm were prepared in adult Sprague−Dawley rat mandibles to assess the bone regeneration potential of mineralized collagen.^5^ The procedures were authorized by the Animal Use and Care Committee of Peking University (LA2014218). IMC (*N* = 10) and EMC (*N* = 10) scaffolds were sterilized with ethylene oxide, immersed in α-MEM (Gibco, USA) containing 1% penicillin/streptomycin overnight at 4 °C, and implanted into defects without loading cells and cytokines. Half of the rats were sacrificed at 1 week after implantation, and the mandibles were harvested for flow cytometry analysis (Accuri-C6, BD Bioscience). The other half of the mandibles were removed at 2 weeks after implantation, fixed in 10% formalin and scanned by a micro-CT system (Skyscan 1174, Bruker, Belgium) at 53 kV and 810 μA. After scanning, the specimens were demineralized in 0.5 mol·L^−1^ EDTA (pH 7.4) for 14–21 days, embedded in paraffin and cut into 5-µm sections. Randomly selected sections were used for H&E staining for neobone formation analysis. The defect area of each slide was observed using a Zeiss light microscope.

### Flow Cytometry

Transplanted scaffolds were harvested from defect area after EMC and IMC post-implantation for 1 week. The scaffolds were minced and cell were flushed with Ca^2+^ and Mg^2+^ free PBS with 2% fetal bovine serum for at least 3 times and then incubated with Anti-CD90 antibody, a surface marker of MSCs, at 1:100 dilution for 30 mins (BD Biosciences). For cell culture, the cells were trypsinized after cultured for 3 days with DMSO or GW4869, and incubated with M2 macrophages antibodies Anti-CD68 (BD Biosciences) and Anti-CD163 (BD Biosciences) at 1:100 dilution for 30 mins. DAPI was used to exclude dead cells. The flow cytometry analysis of positive cells was performed on the Accuri-C6 (BD Bioscience).

### Immunofluorescence staining

Specimens were immersed in antigen retrieval solution for 10 min, blocked with 5% bovine serum albumin for 60 min and incubated overnight at 4 °C with primary antibodies against rat CD163 (1:100, Santa Cruz) as an M2 marker, CD63 (1:500, Abcam) as an sEV marker and CD90 (1:500, CST) as a stem cell surface marker. After rising thoroughly in PBST, goat anti-mouse IgG/RBITC, and goat anti-rabbit IgG/FITC secondary antibodies were applied to the sections for 60 min. The specimens were mounted with DAPI solution and observed with a Zeiss laser-scanning microscope (LSM 510).

### Cell culture and EV isolation

The 6-well plates coated with IMC or EMC films were sterilized using 75% ethanol for 2 h and then under ultraviolet light for 2 h before use. Human THP-1 monocytes (1 × 10^6^) were cultured in 6-well plates with different films and induced to differentiate into macrophages with RPMI 1640 medium containing 10% exosome-free fetal bovine serum (FBS), 2 mM glutamine, antibiotics, and 50 ng·mL^−1^ phorbol myristate acetate (P1585, Sigma, USA) treatment for 24 h at 37 °C. For exosome depletion from FBS, RPMI 1640 containing 20% FBS was centrifuged at 100 000 × *g* for 18 h according to the protocol.^16^ When the cells reached 80%–90% confluence, the supernatant was collected, and total sEVs were isolated from macrophages based on protocols from a previous study.^33^ In brief, the cell culture supernatant was collected and centrifuged at 300 × *g* for 10 min, followed by another centrifugation at 2 000 × *g* for 10 min. After removing non-adherent cells, the supernatant was centrifuged at 10 000 × *g* for 60 min and filtered through a 0.22 μm filter (Millipore, USA) to separate microvesicles. The final supernatant was ultracentrifuged (Beckman Coulter, USA) at 100 000 × *g* for 70 min. The pellet was washed with PBS to eliminate contamination and centrifuged at 100 000 × *g* for another 70 min. Then, EVs were resuspended in PBS and characterized by TEM, nanoparticle tracking analysis, AFM, and Western blotting.

### Transmission electron microscopy

TEM was used to verify the presence of sEVs in the purified samples. Isolated vesicles were fixed in 2% paraformaldehyde and then loaded on a formvar-carbon-coated grid. After washing with PBS, the sEVs were postfixed in 2% glutaraldehyde for 5 min with contrast staining in 2% phosphotungstic acid for 5–10 min. The samples were washed and dried and then examined under an electron microscope (JEM-1400, Japan).

### Nanoparticle tracking analysis

To measure the size distribution of isolated EVs, nanoparticle tracking analysis was performed using the Nanosight system of ZetaView (Particle Metrix, German). After ultracentrifugation, the collected vesicles were suspended in 100 μl PBS, followed by gradient dilution. The samples were loaded into the viewing chamber, and then the vesicles were tracked by microscopy and analyzed with software to measure the size of the vesicles in the samples.

### Atomic force microscopy

The nanostructure and nanomechanical properties of EVs were tested using AFM (Dimension Icon, Bruker, USA) as previously described.^5^ Purified EVs were absorbed to freshly cleaved mica and scanned under peak-force tapping mode with a 1.0 Hz scan rate and a 250 mV amplitude set point. Data were analyzed using Nanoscope Analysis software 1.60. To calculate the Young’s modulus, three scans of representative areas were performed for each specimen. Each scan generated a mapping image with a 512 × 512 resolution for the Young’s modulus. Ten 600 nm × 600 nm samples were randomly selected from the sEV region in each mapping image.

### Western blotting

To measure EV proteins, total proteins were extracted and separated by 10% SDS-PAGE, transferred to a nitrocellulose membrane (Millipore) and blotted with anti-CD63 (1:500, Abcam) and Tsg101 (1:1 000, Abcam), anti-BMP2 (1:500, CST) and anti-GAPDH (1:1 000, Proteintech) antibodies.

To measure endogenous proteins, cell lysate proteins (20 μg) were separated on a 4%–12% SDS polyacrylamide gel. The proteins were extracted by RIPA buffer (Thermo Scientific, Rockford, IL), transferred to PVDF membranes (Invitrogen) and immunoblotted using primary antibodies against GAPDH (1:1 000, Proteintech), BMP2 (1:500, CST), p-Smad3 (1:500, CST), p-Smad5 (1:500, CST), Smad3 (1:500, CST), and Smad5 (1:500, CST). IRDye^®^ 680 or 800CW secondary antibodies (1:10 000, LI-COR, Lincoln, Nebraska) were applied for 30 min. The signals were detected by an Odyssey^®^ Imaging System.

### PKH26-labeled EV transfer

Purified sEVs derived from macrophages cultured on regular plates, EMC and IMC scaffolds were labeled with a PKH26 fluorescent labeling kit (Sigma-Aldrich, MO, USA) as previously reported.^40^ sEVs were incubated with 2 μmol·L^−1^ PKH26 for 5 min and then washed five times using a 100-kD filter (Millipore) to remove excess dye. sEVs were harvested by using an exosome isolation reagent (Invitrogen) according to the manufacturers’ instructions. Labeled sEVs were incubated with human bone marrow MSCs for 4 h. MSCs were then fixed with formaldehyde and stained with phalloidin for 30 min. Images were observed using a Zeiss laser-scanning microscope (LSM 510).

### Quantitative real-time polymerase chain reaction (PCR)

To investigate whether macrophages in response to scaffolds could regulate MSC osteogenesis, human bone marrow MSCs were cultured with regular medium supplemented with macrophage-derived sEV supernatant at a ratio of 1:1 for 14 days. Total RNA was isolated from the primary cells and tendon tissues using TRIzol reagent (Thermo Fisher Scientific) according to the manufacturer’s instructions. Then, the mRNA was converted to complementary DNA. Real-time PCR was carried out using gene-specific primers and SYBR Green (Invitrogen) on 7900HT Fast Time PCR. The primer sequences were as follows: ALP-F, 5′-ACTTCCAGACCATTGGCTTG-3′ and ALP-R, 5′-TTCTTGGCCCGATTCATCAC-3′; BGLAP-F, 5′-GTGCAGCCTTTGTGTCCAAG-3′, and BGLAP-R, 5′-TCCGGATTGAGCTCACACAC-3′; BMP2-F, 5′-TGCACCAAGATGAACACAGC-3′ and BMP2-R, 5′-TTCCGCTGTTTGTGTTTGGC-3′; COL1A1-F, 5′-AGACGAAGACATCCCACCAATC-3′, and COL1A1-R, 5′-ATCACGTCATCGCACAACAC-3′; OSX-F, 5′-AAGTTCACTATGGCTCCAGTCC-3′, and OSX-R, 5′-TTCTTTGTGCCTGCTTTGCC-3′; RUNX2-F, 5′-AAGGCACAGACAGAAGCTTG-3′, and RUNX2, 5′-AGGAATGCGCCCTAAATCAC-3′; and GAPDH-F, 5′-AATTCCATGGCACCGTCAAG-3′ and GAPDH-R, 5′-ATCGCCCCACTTGATTTTGG-3′. The expression levels were calculated by using the GAPDH expression level as an internal control. The 2^−^^▵▵^^Ct^ method was used to quantify the relative gene expression levels.

### Alizarin Red S staining

To detect mineral nodule formation, human bone marrow MSCs were cultured in osteogenic medium supplemented with the collected sEV supernatant at a ratio of 1:1 for 14 days. After removing the medium, MSCs were rinsed with ddH_2_O, fixed in 4% paraformaldehyde, and stained with 2% Alizarin Red S. The stained MSCs were observed using a Zeiss light microscope and quantified by optical density measurement at 562 nm.

### Extracellular vesicle neutralization by GW4869

Human THP-1 monocytes were cultured on IMC-coated 6-well culture plates and induced to differentiate into macrophages by phorbol myristate acetate treatment for 24 h at 37 °C. After THP-1-derived macrophage adherence, the cells were cultured in RPMI 1640 medium and supplemented with 10% EV-free FBS for 48 h with 10 μmol·L^−1^ GW4869 or DMSO. Then, the medium was collected, centrifuged at 2 000 × *g* for 10 min, and filtered with a 0.22-μm filter (Millipore, MA, USA) to remove cell debris. When human bone marrow MSCs were treated, the collected conditioned medium was added to regular or osteogenic medium at a ratio of 1:1. MSC proliferative, immunomodulative and osteogenic potential were tested by CCK-8, flow cytometry and real-time PCR, respectively, as stated before. The protein levels of BMP2 and Smad5 were also evaluated by Western blotting as described before.

### Statistical analysis

All data are presented as the mean ± standard deviation and assessed for significance by a two-tailed independent Student’s *t*-test or by one-way analysis of variance. Differences with *P* < 0.05 were considered statistically significant.

## Supplementary information

Supplementary File

## Data Availability

All data associated with this study are presented in the paper.
