# Supplementary material for: Macrophage-derived small extracellular vesicles promote biomimetic mineralized collagen-mediated endogenous bone regeneration
Source: Int J Oral Sci. 2020 Nov 30;12:33. doi: 10.1038/s41368-020-00100-6 (PMC7705747; doi:10.1038/s41368-020-00100-6)
Supplement: Supplementary file 1 — Supplementary File [file 41368_2020_100_MOESM1_ESM.docx]

**Macrophage-derived small extracellular vesicles promote biomimetic mineralized collagen-mediated endogenous bone regeneration**

An-Qi Liu^1^, Shanshan Jin^2^, Cui-Cui Fu^3^, Shengji Cui^2^, Ting Zhang^2^, Lisha Zhu^2^, Yu Wang^2^, Steve GF Shen1^,4*^, Nan Jiang^5*^, Yan Liu^2*^

^1^Department of Oral and Maxillofacial Surgery, Ninth People’s Hospital, Shanghai Jiao Tong University School of Medicine, Shanghai Key Laboratory of Stomatology, Shanghai 200011, China

^2^Laboratory of Biomimetic Nanomaterials, Department of Orthodontics, Peking University School and Hospital of Stomatology, National Engineering Laboratory for Digital and Material Technology of Stomatology, Beijing Key Laboratory of Digital Stomatology, Beijing 100081, China

^3^Department of Oral Biochemistry, Academic Centre for Dentistry Amsterdam (ACTA), University of Amsterdam (UvA) and Vrije Universiteit Amsterdam (VU), Gustav Mahlerlaan 3004, 1081 LA Amsterdam, The Netherlands

^4^Shanghai University of Medicine and Health Sciences, Shanghai 201318, China

^5^Central Laboratory, Peking University School and Hospital of Stomatology, National Engineering Laboratory for Digital and Material Technology of Stomatology, Beijing Key Laboratory of Digital Stomatology, Beijing 100081, China

*Corresponding author E-mail: shengf@sumhs.edu.cn (S.S.); [nanjiang@bjmu.edu.cn](mailto:nanjiang@bjmu.edu.cn) (N.J.); [orthoyan@bjmu.edu.cn](mailto:orthoyan@bjmu.edu.cn) (Y.L.)

**Materials and Methods**

**Western blotting**

To measure EVs’ proteins, total proteins were extracted from secreted EVs. Proteins were separated by SDS-PAGE and transferred to a nitrocellulose membrane (Millipore) and blotted with anti-CD63 antibodies (1:500, abcam), anti-BMP2 (1:500, CST) and anti-GAPDH (1:1000, Proteintech).


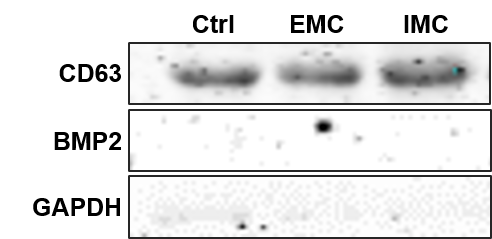
**Supplementary Fig. 1** Western blotting of macrophages derived EVs’ proteins.
